# Pharmacophore-guided repurposing of fibrates and retinoids as GPR40 allosteric ligands with activity on insulin release

**DOI:** 10.1080/14756366.2020.1864629

**Published:** 2021-02-02

**Authors:** Erika Cione, Maria Cristina Caroleo, Hiroyuki Kagechika, Fabrizio Manetti

**Affiliations:** a Department of Pharmacy, Health and Nutritional Sciences (Department of Excellence 2018-2022), University of Calabria, Rende, Italy; b Institute of Biomaterials and Bioengineering, Tokyo Medical and Dental University (TMDU), Tokyo, Japan; c Department of Biotechnology, Chemistry and Pharmacy (Department of Excellence 2018-2022), University of Siena, Siena, Italy

**Keywords:** Virtual screening, pharmacophore model, drug repurposing, GPR40, insulin secretion

## Abstract

A classical drug repurposing approach was applied to find new putative GPR40 allosteric binders. A two-step computational protocol was set up, based on an initial pharmacophoric-based virtual screening of the DrugBank database of known drugs, followed by docking simulations to confirm the interactions between the prioritised compounds and GPR40. The best-ranked entries showed binding poses comparable to that of TAK-875, a known allosteric agonist of GPR40. Three of them (tazarotenic acid, bezafibrate, and efaproxiral) affect insulin secretion in pancreatic INS-1 832/13 β-cells with EC_50_ in the nanomolar concentration (5.73, 14.2, and 13.5 nM, respectively). Given the involvement of GPR40 in type 2 diabetes, the new GPR40 modulators represent a promising tool for therapeutic intervention towards this disease. The ability to affect GPR40 was further assessed in human breast cancer MCF-7 cells in which this receptor positively regulates growth activities (EC_50_ values were 5.6, 21, and 14 nM, respectively).

## 1. Introduction

Drug repurposing or drug repositioning approaches have been applied in recent years with the aim of facilitating the drug discovery process. In fact, existing drugs or drug candidates, whose ADME-Tox properties have been already explored by previous research, can be moved towards new possible therapeutic applications. Following this way, the time- and money-consuming steps of the classical drug design process to access and pass phases of clinical trials are significantly alleviated^1^^,^^2^.

The seven-transmembrane receptor GPR40 (FFA1/FFAR1) is highly expressed in pancreatic β-cells as well as in intestinal epithelium and is activated by physiological concentrations of both free and conjugated fatty acids^3–5^. Activation of GPR40 enhances glucose-stimulated insulin secretion (GSIS) but does not affect insulin secretion at low glucose concentrations^6^. Enhancement of GSIS by GPR40 has been confirmed *in vitro*, in INS-832/13 cell line harbouring human insulin clone^4^^,^^5^, *in vivo* and in clinical trials^7^^,^^8^. These considerations led to the conclusion that agonists of the GPR40 receptor can be profitable tools for the treatment of type 2 diabetes (T2D) and related diseases^9^.

The most intriguing compound able to act as an allosteric agonist of GPR40 is TAK-875^10^ that accessed phase III clinical trials as an antidiabetic and antihyperglycemic agent. However, liver toxicity caused the termination of clinical studies, although the significant beneficial effects in diabetic patients. Fortunately, an in-depth analysis of experimental results on TAK-875 strongly suggested that compound attrition was caused by off-target effects (in particular, inhibition of hepatobiliary transporters) and was independent of targeting GPR40. These considerations have consolidated the foundations of further development of GPR40 agonists^11^.

Therefore, to identify new small molecules able to modulate the activity of GPR40, a pharmacophore-based virtual screening approach, refined by molecular docking simulations, has been applied to chemical databases of known drugs or compounds in the clinical phase to possibly reposition several database entries as GPR40 allosteric ligands.

The best-scored compounds resulting from the two-step pharmacophore- and docking-based virtual screening protocol underwent biological tests using INS-1 832/13 cells as an *in vitro* model of insulin secretion. The compounds’ ability to affect GPR40 was further assessed in a human breast cancer cell line (MCF-7) in which this receptor positively regulates growth activities^12^^,^^13^.

The biological assays showed that two propanoic acid derivatives of the fibrate class of compounds (namely, bezafibrate and efaproxiral) and the acetylenic retinoid derivative tazarotenic acid were active as GPR40 agonists, thus supporting the computational results.

## 2. Material and methods

### 2.1. Chemicals and instruments

The retinoic acid receptors (RARs) pan-antagonist 4-(13*H*-10,11,12,13-tetrahydro-10,10,13,13,15 pentamethyldinaphtho[2,3-*b*][1,2-*e*][1,4]diazepin-7-yl)benzoic acid (LE540) and the retinoid X receptors (RXRs) pan-antagonist 4-(7,7,10,10-tetramethyl-5-propyl-7,8,9,10-tetrahydro-5*H*-benzo[*b*]naphtho[2,3-*e*][1,4]diazepin-12-yl)benzoic acid (HX-603) were synthesised according to previous literature^14–16^ and dissolved in DMSO. DMSO and 3-(4,5-dimethylthiazol-2-yl)-2,5-diphenyltetrazolium bromide (MTT) were purchased from Sigma Aldrich (St. Louis, MO). A microtitre plate reader (Synergy H1 by BioTek, Winooski, VT) was used for the biological assays.

### 2.2. Biological assays

#### 2.2.1. Cell Cultures

The clonal rat INS-1 832/13 β-cell line (a gift from Dr. C. Newgard, Duke University, Durham, NC) was maintained in RPMI 1640 with 10% foetal bovine serum (FBS), 11 mM glucose, 10 mM HEPES, 2 mM L-glutamine, 1 mM sodium pyruvate, 50 µM β-mercaptoethanol, 100 U penicillin/mL, and 100 U streptomycin/mL (Thermofisher, Milan, Italy).

Human breast adenocarcinoma MCF-7 (ERα-positive) cell line was grown in Dulbecco’s modified Eagle’s medium-F12 plus glutamax containing 5% FBS and 1 mg/mL penicillin-streptomycin (Thermofisher). According to our previous protocols, both cell lines were incubated in 5% CO_2_ at 37 °C. Media were refreshed every 2–3 days. Cells were sub-cultured when reached >70% confluence. The cell lines used were kept without serum during the experimental procedure to avoid endogenous retinoid synthesis^17^.

#### 2.2.2. Glucose stimulating insulin secretion (GSIS) from INS-1 832/13 β-cells

Pancreatic INS-1 832/13 β-cells were plated in 24 wells plates with RPMI-1640, 11 mM glucose, 10% FBS at 5 × 10^5^ cells mL^−1^ density. The next day, the medium was switched to 5 mM glucose and 10% FBS. After 16 h incubation, cells were washed with phosphate-buffered saline (PBS) and the GSIS was performed. In the GSIS, secretion media HBSS with 20 mM Hepes and 1% BSA, pH 7.2, containing 3 mM glucose, was added to the cells in presence of double IC_50_ concentration of RAR and RXR antagonists (LE540 and HX-603, respectively), diluted in DMSO. DC260126 (12 µM) was added 30 min before treatments in HBSS 3 mM glucose (the final DMSO concentration was 0.03%). After 2 h, the HBSS was replaced by fresh secretion media HBSS containing 23 mM glucose. GSIS was performed with the test compounds tazarotenic acid, efaproxiral, and bezafibrate (1–100 nM) for 5 min incubation. The amount of insulin in the cell medium was analysed by insulin ELISA kit (Calbiotech Inc) in agreement with the manufacturer’s instructions and our previous work^18^. Briefly, the medium was spun 5 min at 2,500 rpm, 4 °C, to pellet down cellular debris and immediately assayed or frozen in liquid N_2_ first and −80 °C successively, for no longer than 1 week.

#### 2.2.3. Cell growth assay on MCF-7 cells

Selected compounds (tazarotenic acid, efaproxiral, and bezafibrate) were tested for *in vitro* activity in MCF-7 cell line, using the MTT assay. The MCF-7 cells were seeded in a 96-well plate (1 × 10^3^ cells/well) and then incubated at 37 °C under 5% CO_2_ for 24 h. Pre-treatment with double IC_50_ concentration of RAR and RXR antagonists diluted in DMSO, was added 120 min before treatments. DC260126 (a GPR40 antagonist, 12 µM) was added 30 min before treatments (the final DMSO concentration was 0.03%). Stock solutions of each compound were then added to the plates with concentrations ranging from 1 nM to 100 µM (1:10^5^ dilutions) and the plates were incubated for 24 h. Cell viability was then measured by the MTT assay that is based on the reduction of the tetrazolium salt by live cells during growth^19^. Absorbance was read at 570 nm, and the EC_50_ values were calculated using GraphPad Prism v 5.0.0 software (GraphPad Software Inc., La Jolla, CA).

#### 2.2.4. Statistical analysis

Results were expressed as mean ± SD from three independent experiments. Statistical differences were determined by one-way analysis of variance (ANOVA) using Graph Pad 5.0 software followed by Tukey’s method. Differences were considered statistically significant for *p* < 0.05 (*), *p* < 0.01 (**), *p* < 0.001 (***).

### 2.3. Computational details

The structure of GPR40 co-crystallised with TAK-875 (resolution 2.33 Å, protein data bank entry 4phu)^20^ was refined by application of the Protein Preparation Wizard (software Maestro, release 2019-1)^21^ to assign bond orders, add hydrogen atoms, adjust missing side chains, cap termini, and generate tautomeric states. Next, the Develop Pharmacophore Model routine of the software Phase was applied to the GPR40/TAK-875 complex to generate automatically a pharmacophoric model that comprised excluded volumes. The resulting model was constituted by five features: one negative ionic group, two hydrophobics, and two aromatic rings. On the other hand, the sd file containing all the about 14,000 compound structures stored in the DrugBank database^22^ was downloaded and converted into a Phase database. For each compound, possible ionisation and tautomeric states were generated by Epik software (pH ranging from 6.0 to 8.0), together with 200 conformations. Phase was also used to filter the DrugBank database with the pharmacophoric model, using default screening settings. As a result, the resulting 36 putative hit compounds were ranked by decreasing Phase Screen Score, from 0.98 to 0.84. Expectedly, the best-ranked compound found by the pharmacophore-based virtual screening was TAK-875, thus suggesting that the entire computational protocol could be a reliable tool for the identification of new GPR40 allosteric ligands. The next positions of the ranking list were occupied by tazarotenic acid, efaproxiral, and bezafibrate. To find their binding pose and interaction pattern with GPR40, such compounds were submitted to docking simulations (software Glide) into the TAK-875 binding site. For this purpose, the shape and properties of the receptor were codified within a grid that was built around the co-crystallised ligand. Rotation of hydroxyl and thiol groups of GPR40 included in the grid box was allowed during grid generation. The resulting receptor grid was then used to find suitable positions for docked ligands and score the ligand/GPR40 interactions found in that pose. The reliability of Glide-based docking calculations on the allosteric binding site of TAK-875 was previously verified^23^.

Molecular dynamics simulations were performed to check for the stability of the docked complexes. Before simulations, docking complex between GPR40 and tazarotenic acid (taken as a representative example) was solvated in a box of explicit water molecules containing counterions to neutralize the charge. Subsequently, the overall system was relaxed through energy minimization, heated to 300 K and equilibrated to finally produce a 100 ns MD trajectory.

The three putative new GPR40 allosteric ligands were purchased by chemical vendors (tazarotenic acid: Cayman Chemical, item no. 21367, purity ≥ 98%; efaproxiral: MedChemExpress, cat. no. HY-13619, purity ≥ 99.9%; bezafibrate: Cayman Chemical, item no. 10009145, purity ≥ 98%) and submitted to biological evaluation.

## 3. Results and discussion

### 3.1. Virtual screening based on a pharmacophoric model and molecular docking simulations

**Figure 1.**
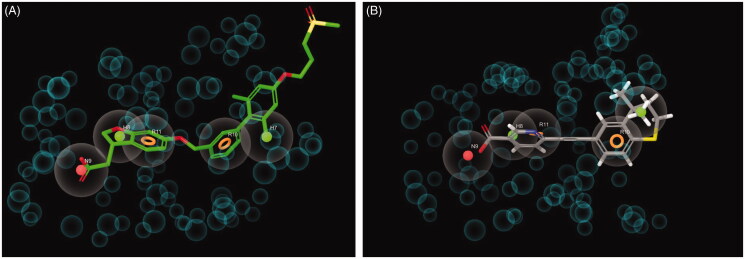
Graphical representation of the interaction pattern between the five feature pharmacophoric model for GPR40 allosteric ligands and the co-crystallized TAK-875 (A) or tazarotenic acid (B) that was identified by a pharmacophore-based virtual screening approach. N9 (the orange sphere) is a negative ionic group; H7 and H8 (the green spheres) are hydrophobics; R10 and R11 (the orange circles) are aromatic rings. Large grey spheres are regions of space where the chemical group corresponding to the pharmacophoric feature can be accommodated. On the contrary, small grey spheres represent regions of space where parts of the macromolecule are located and that are forbidden to ligands (excluded volumes).

**Figure 2.**
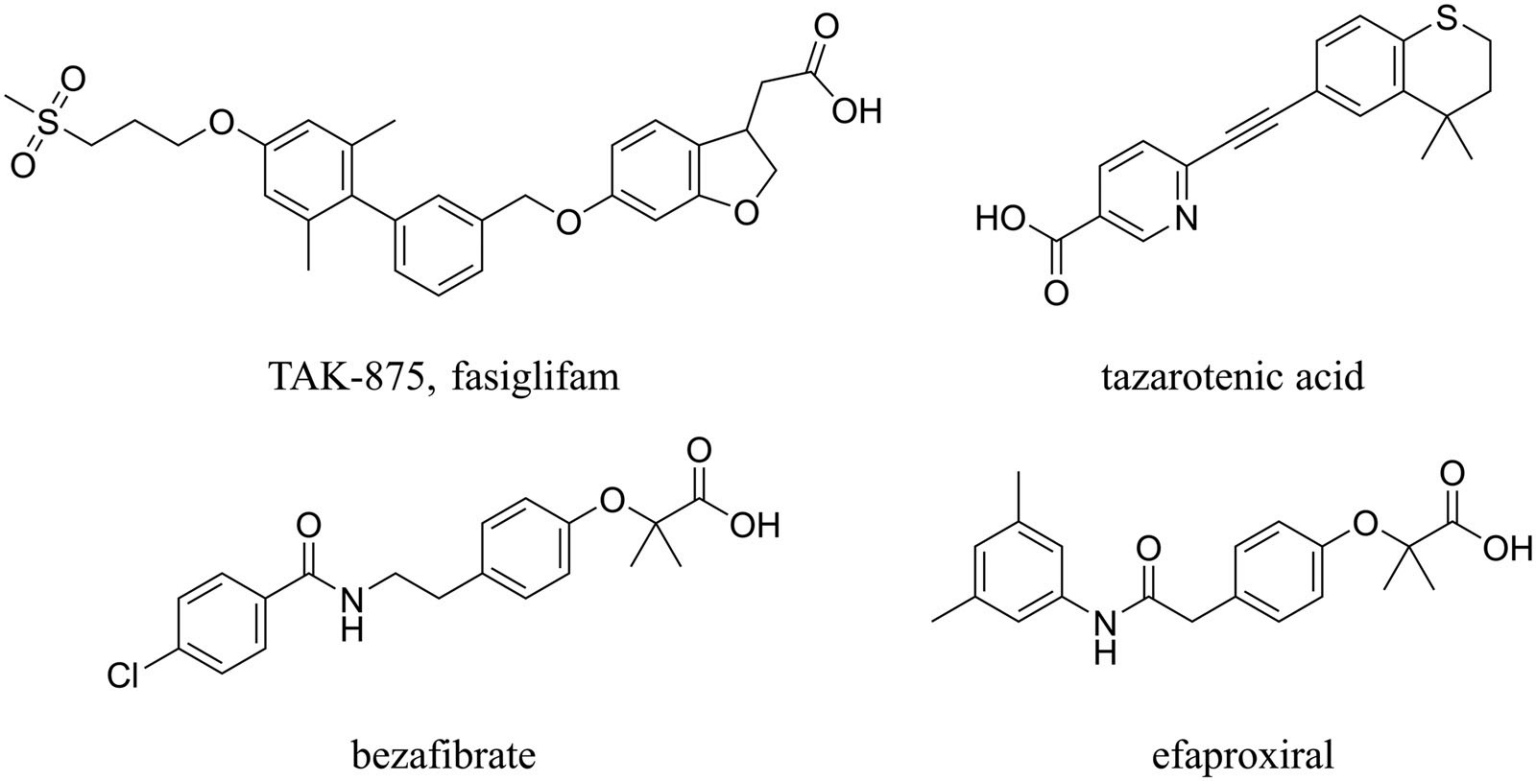
Structure of TAK-875 (the co-crystallized ligand of GPR40), the diarylethynyl derivative tazarotenic acid, and the two phenoxyacetic derivatives belonging to the fibrate class of compounds.

The X-ray crystallographic three-dimensional coordinates of the structure of human GPR40 bound to allosteric agonist TAK-875 (also known as fasiglifam, Figure 2) were used to set up a virtual screening approach aimed at finding a new putative allosteric ligand of the target. For this purpose, a three-dimensional pharmacophoric model was generated automatically by Phase software^24^ taking into account complementarity between chemical features of both TAK-875 and GPR40. Excluded volumes, which represent regions of space occupied by portions of GPR40 and thus forbidden to putative ligands, were also added to the model. The resulting pharmacophore was constituted by five chemical features (Figure 1(A)): a negative ionic group (N9) corresponding to the terminal carboxyl substituent of TAK-875, two hydrophobics (H8 and H7, respectively) that represent the dihydrofuran ring and one of the two methyl groups of the xylenyl moiety, and two aromatic ring features (R11 and R10, respectively) that codify for the presence of the condensed and the central phenyl rings of TAK-875. The terminal methanesulfonylpropoxy chain of the ligand, which was exposed to the solvent without interactions with GPR40, did not contribute to generating portions of the pharmacophoric model.

Next, following a classical drug repositioning or repurposing approach often applied in medicinal chemistry projects, the pharmacophore was used as a three-dimensional filter to screen in silico the DrugBank database^22^. The latter is a bioinformatics and cheminformatics database that contains about 14,000 entries mainly including approved and discovery-phase drugs. Within the virtual screening procedure, the Phase ligand screening routine was applied, forcing the software to keep only those compounds able to match, with their chemical groups, all the five features of the pharmacophore. As a result, a list of 36 putative new GPR40 allosteric ligands was generated. Among them, as expected, TAK-875 was found as the best-ranked hit compound, thus suggesting that the pharmacophore is a reliable model for identifying new allosteric ligand of GPR40. Additional three compounds (Figure 2) occupied the first positions of the ranking list: tazarotenic acid, an acetylenic derivative belonging to the retinoid class of compounds, which is reminiscent of GPR40 agonists bearing a diaryl ethynyl scaffold, such as TUG-424^25^ and similar compounds; bezafibrate and efaproxiral, two propanoic acid derivatives belonging to the fibrate class of compounds that share their phenoxyacetic acid substructure with many GPR40 ligands recently appeared in the literature^26–30^. These three compounds showed a very good match with the pharmacophore. As an example, tazarotenic acid mapped the negative ionic region N9 with its carboxyl terminus (Figure 1(B)), while the two aromatic ring regions R11 and R10 were filled by the pyridine and the condensed phenyl ring, respectively. Moreover, one of the methyl groups of the *gem*-dimethyl moiety of the dihydrothiochromenyl nucleus mapped the hydrophobic region H7. Finally, the hydrophobic region H8 was filled by part of the pyridine nucleus. It is worth noting that, differently from many GPR40 agonists that suffered from high molecular weight and lipophilicity^29^, the three compounds showed molecular weight values between 323 (tazarotenic acid) and 362 (efaproxiral), while their clogP values (calculated with ChemAxon software) were in the range between 3.52 (bezafibrate) and 4.33 (tazarotenic acid). The corresponding values for TAK-875 were 525 and 3.99, respectively. Moreover, tazarotenic acid, efaproxiral, and bezafibrate share the additional advantage to be achiral molecules.

**Figure 3.**
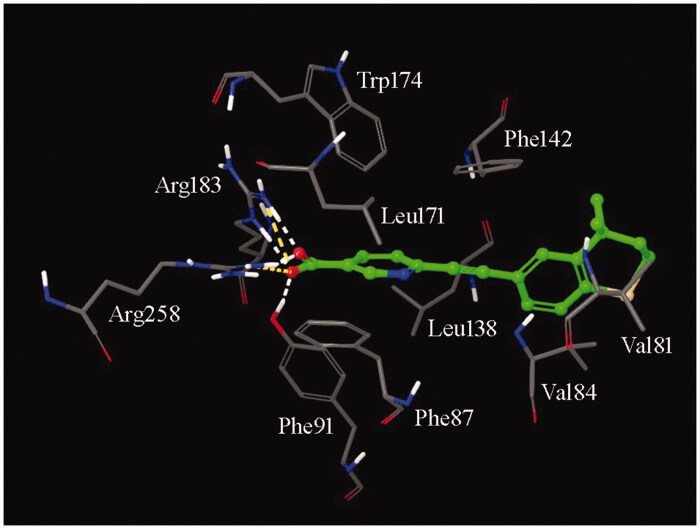
Graphical interaction of the interaction pattern of tazarotenic acid (ball and stick notation, green) within the allosteric binding site of GPR40 (for the sake of clarity, only a few amino acids are displayed). The carboxylate group serves as anchor point of the ligand by binding the guanidino side chains of Arg183 and Arg258, as well as the phenolic substituent of Phe91. The central pyridine ring makes hydrophobic interactions with the aromatic side chains of Phe87 and Trp174, as well as with the alkyl side chain of Leu171. Finally, the terminal condensed moiety points towards the solvent interface and shows additional hydrophobic contacts with Phe142, Leu138, Val84, and Val81.

To further check the hypothesis that such compounds could act as GPR40 allosteric ligands, they were submitted to molecular docking calculations to identify their binding pose within the allosteric binding site on GPR40. As an example of the results, the carboxylate oxygens of tazarotenic acid in its best orientation made hydrogen bonds and salt bridges with the side chain of Arg183 and Arg258, as well as with the phenolic substituent of Phe91 (Figure 3). The pyridine ring is embedded within a lipophilic cage delimited by Phe87, Leu171, and Trp174, while the terminal dihydrothiochromenyl edge contacted Val81, Val84, Leu138, and Phe142.

MD simulations could offer preliminary information about the time-dependent stability of final complexed states of these three new binders. To assess the stability of the docked complexes and to obtain further information about ligand-protein interactions, MD simulations were performed by using Desmond software. The evolution of the RMSD calculated on the ligand atoms of the complex during MD simulation time with respect to the last frame of minimization showed a 0.94 Å mean value with a 0.2 standard deviation, thus suggesting a significant stability of the complex.

The production phases of the MD trajectories were then submitted to cluster analysis to evaluate the most frequent interactions made by the compound within the binding sites. An extended network of both hydrogen bonds and salt bridges was confirmed between the terminal carboxylate moiety and the side chains of Arg183 and Arg258. An additional anchor point consisting in a parallel ρ stacking was found between the pyridine ring of the ligand and the side chain of Phe87.

Upon prioritisation of the first ranked compounds, the final step of the virtual screening protocol was their purchase from chemical vendors and submission to biological assays.

### 3.2. Insulin secretion from INS-1 832/13 β-cells

Compounds prioritised with the virtual screening procedure were dissolved in DMSO and evaluated for their activity on insulin secretion. This assay measures insulin release under GSIS conditions. The functional tests were carried out using one of the most physiologically relevant *in vitro* β-cell models for studying human insulin secretion. The INS-1 832/13 cell line is a genetically modified INS-1 cell subclone selected for its robust glucose responsiveness over the physiological range of glucose concentrations (3–23 mM) retaining a differentiated cell phenotype over more than 6 months in culture. These characteristics made this cell line a widely used tool for studying various aspects of β-cell function and are advantageous in compound screening approaches. Therefore, the molecules were screened for insulin secretion. To avoid interferences with activation of RAR and RXR by tazarotenic acid and bezafibrate (this latter indirectly since the inhibition involved its obligate RXR partners), the cell line was exposed to specific antagonists as pre-treatment for 120 min. Insulin levels were measured in conditioned medium of INS-1 832/13 β-cell line treated with vehicle or various concentrations (from 0 to 100 nM) of tazarotenic acid, efaproxiral, and bezafibrate by two-site immunoassay.

**Figure 4.**
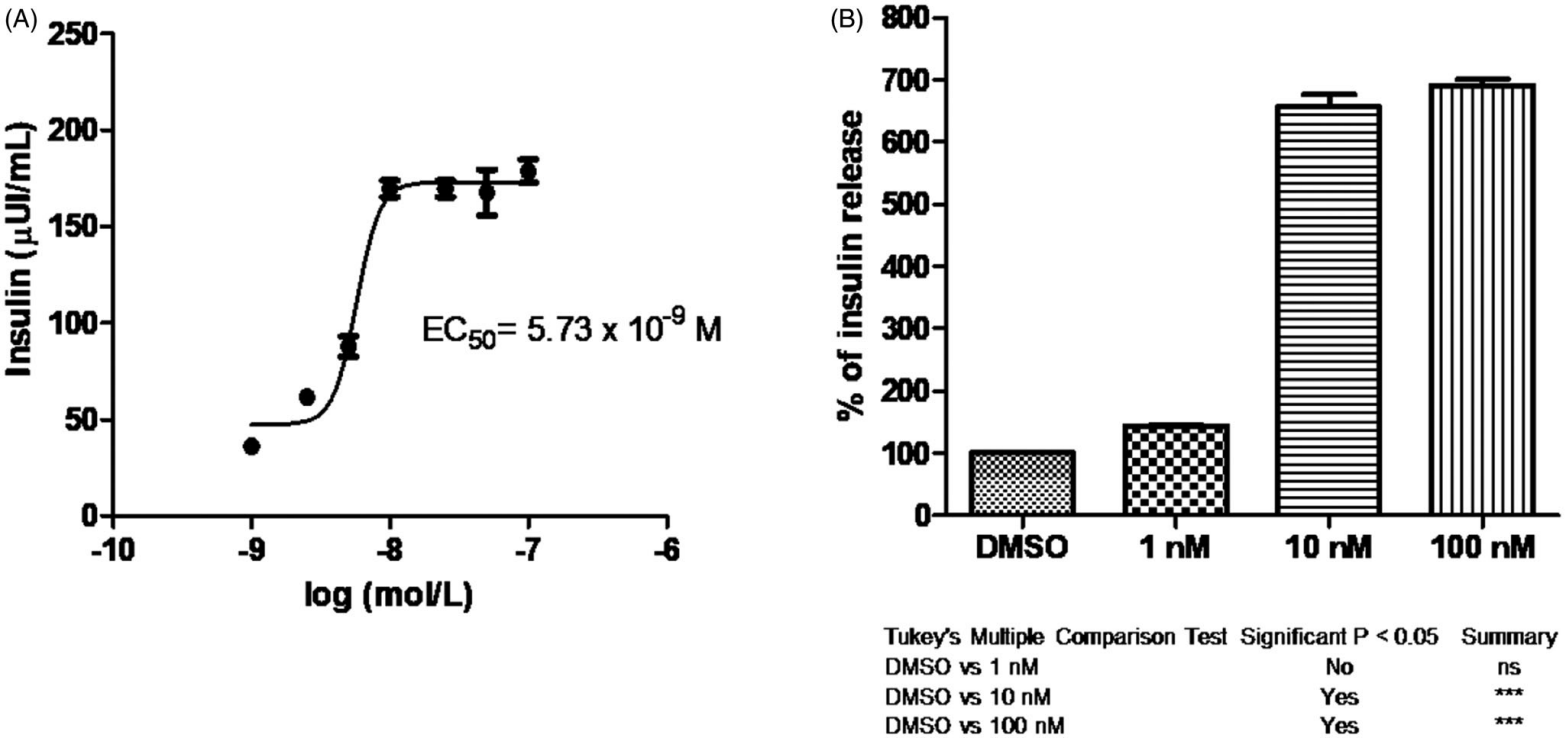
(A) Dose response curve of tazarotenic acid and its EC_50_ value. (B) Percentage of insulin release at different concentrations. Tukey’s multiple comparison test is shown.

Tazarotenic acid, a third-generation acetylenic retinoid, is the drug generated by hydrolytic cleavage of the corresponding ethyl ester prodrug tazarotene. It has been approved for dermatologic use^31^, in topical applications for the treatment of psoriasis and acne. This retinoid showed the best activity on insulin secretion with an EC_50_ = 5.73 nM (the dose-response graph is shown in Figure 4(A)), similar to that found in cell growth assay on MCF-7 cells (5.6 nM, Table 1). There was a corresponding significant dose-dependent increase in insulin secretion percentage, confirmed by Tukey’s multiple comparison test to control (Figure 4(B)).

**Table 1.** Cell viability assay (MTT) in MCF-7 cells.

Compound	EC_50_ (nM)
Tazarotenic acid	5.6
Efaproxiral	21
Bezafibrate	14

In terms of concentration, these data are similar to that of the clinical bioavailability of tazarotenic acid in trans-epidermal administration (from 2 to 5.4 ng/mL)^32^.

In a similar way, the two propionic acid derivatives efaproxiral and bezafibrate were found to increase insulin secretion in a dose-dependent manner. Efaproxiral is an investigational drug analogue of bezafibrate and binds to haemoglobin subunits^33^. It was applied as radio sensitiser in radiation anticancer therapy because of the non-covalent binding to haemoglobin results in facilitating the release of oxygen from the haemoglobin/oxygen complex, thus increasing the oxygen level in tumour cells (i.e. crucial for the effectiveness of radiation therapy), independently from blood–brain barrier penetration. However, results from a phase III trial led to study discontinuation^34^.

On the other hand, bezafibrate is a second line antilipemic agent that belongs to the class of phenoxyacetic acid derivatives, and targets peroxisome proliferator-activated receptor (PPAR) isoforms^35^.

**Figure 5.**
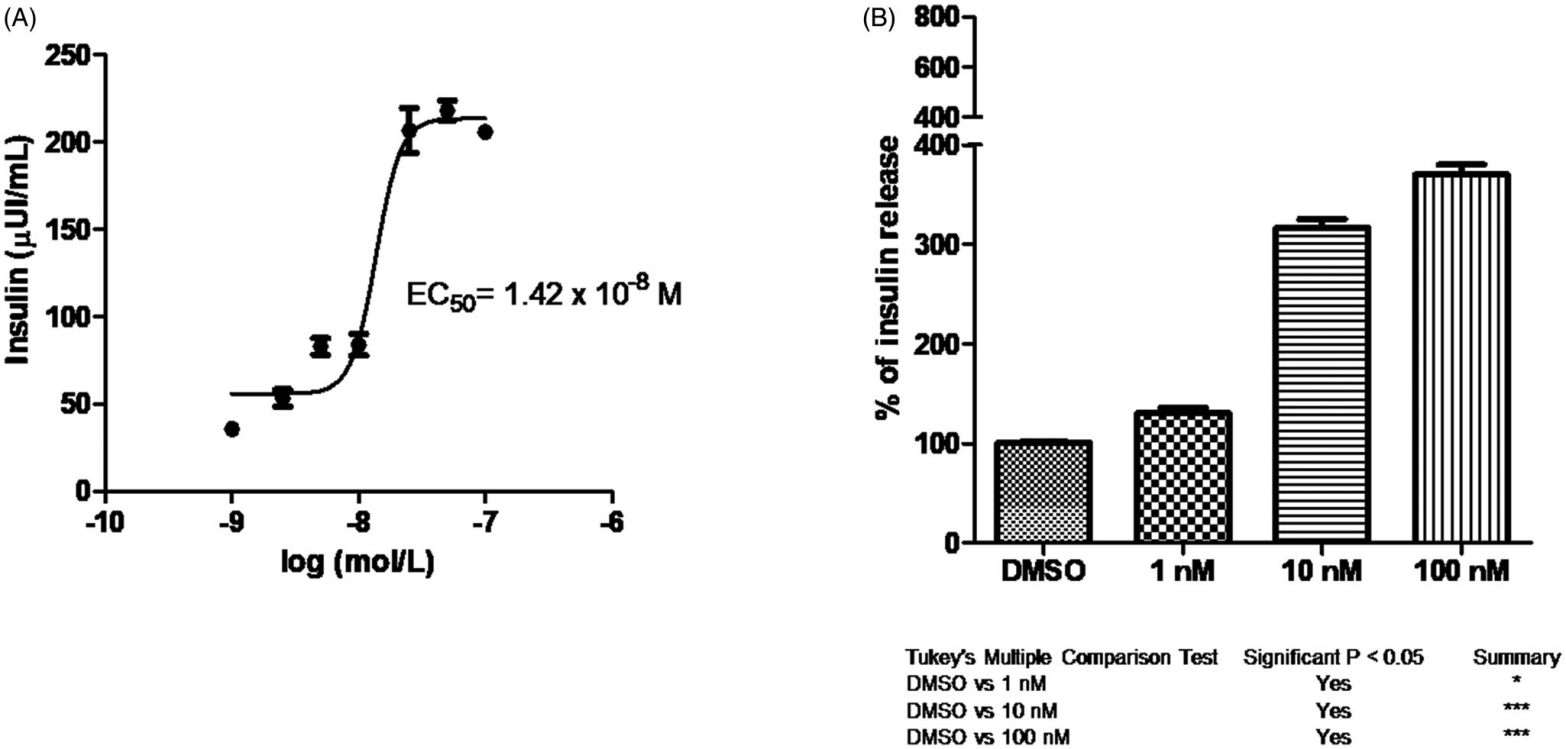
(A) Dose response curve of efaproxiral and its EC_50_ value. (B) Percentage of insulin release at different concentrations. Tukey’s multiple comparison test is shown.

**Figure 6.**
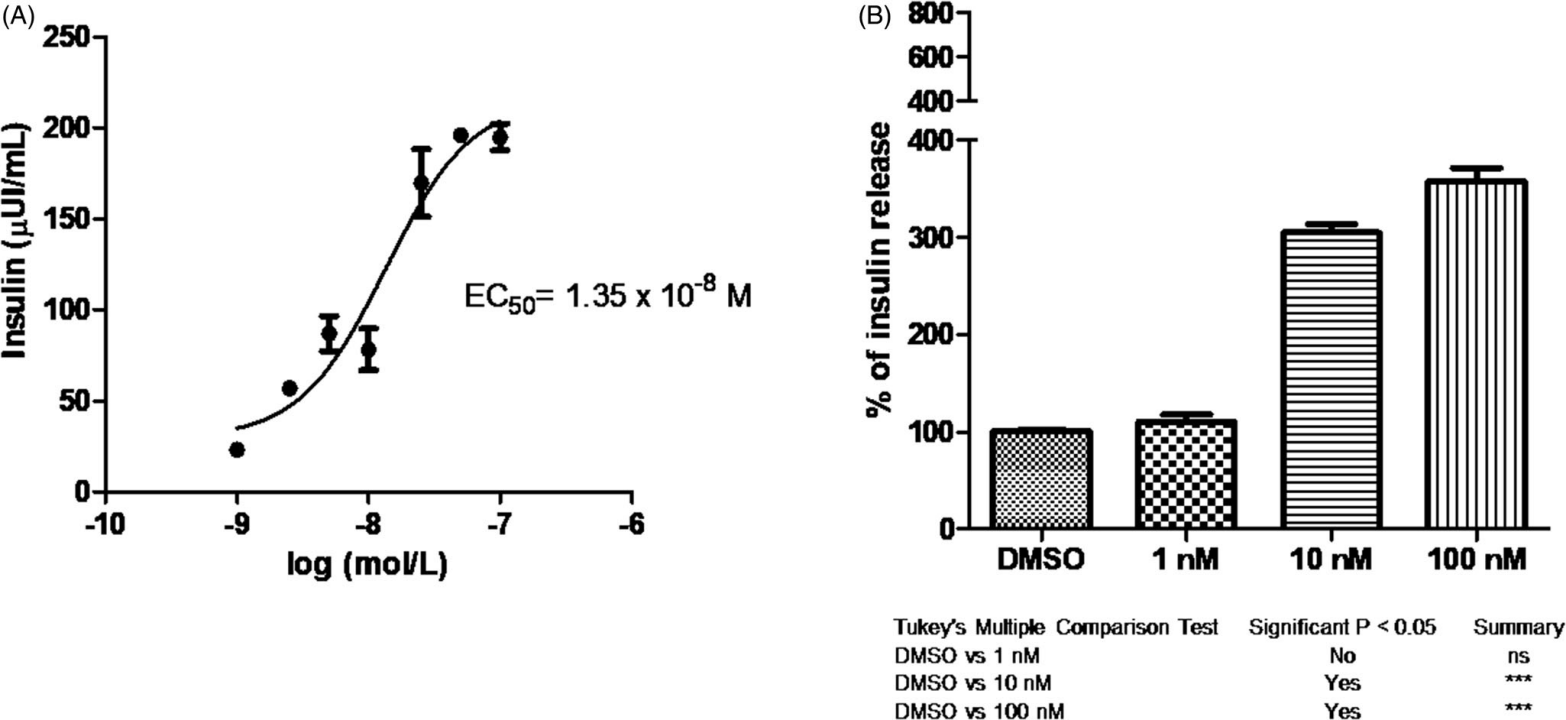
(A) Dose response curve of bezafibrate and its EC_50_ value. (B) Percentage of insulin release at different concentrations. Tukey’s multiple comparison test is shown.

Both efaproxiral and bezafibrate showed a similar profile and EC_50_ values (14.2 nM, Figure 5(A), and 13.5 nM, Figure 6(A), respectively). There was a corresponding significant dose-dependent increase in insulin secretion percentage caused by the two compounds and confirmed by Tukey’s Multiple Comparison Test in comparison to control (Figures 5(B) and 6(B), respectively).

**Figure 7.**
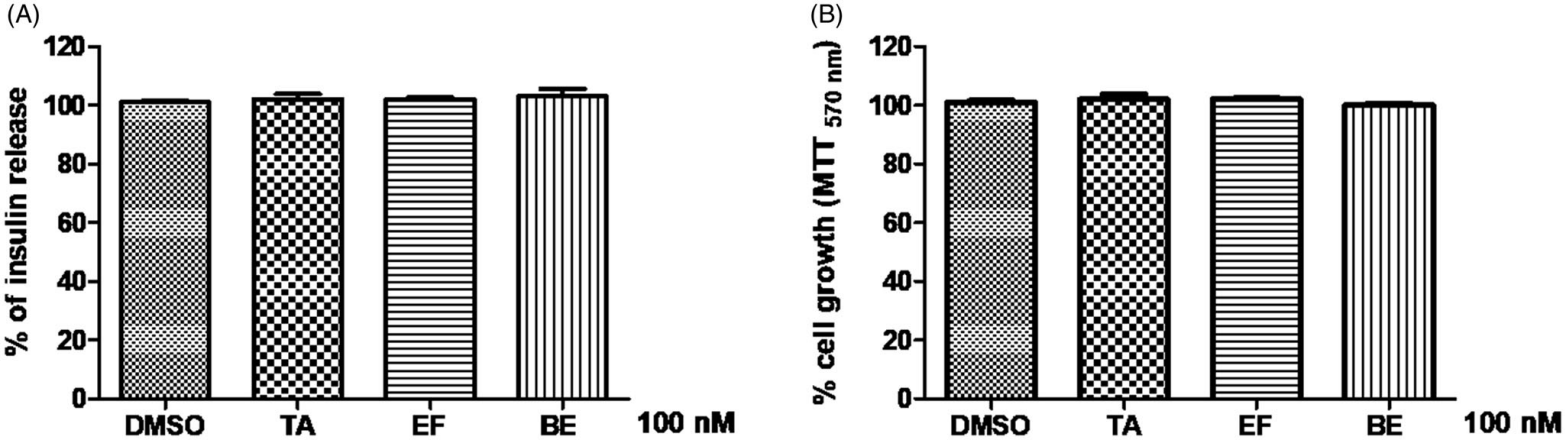
(A) Insulin release and (B) cell growth assay with 100 nM tazarotenic acid, efaproxiral, and bezafribate, respectively, in presence of GPR40 antagonist DC260126.

Overall, experimental evidence strongly indicated that tazarotenic acid, efaproxiral, and bezafibrate acted by inducing cell growth and enhancing insulin secretion. To validate GPR40 as a target for the compounds under investigation, pre-treatment with the known GPR40 antagonist DC260126 (12 μM) abolished cell response, in terms of either insulin release or cell growth (Figure 7(A,B), respectively). In fact, the results of treatment with each of the three compounds (100 nM) were comparable with those of a DMSO treatment.

It is worth noting that all three compounds herein tested accessed clinical trials. Tazarotenic acid and bezafibrate passed to market for the treatment of acne and hyperlipidaemia, respectively. On the contrary, the development of efaproxiral was discontinued upon discouraging results from a phase III clinical trial. In addition, a phase III clinical trial started very recently (March 16, 2020)^36^ where bezafibrate is evaluated for primary sclerosing cholangitis which is an additional T2D risk factor^37^.

### 3.3. Cell growth assay in MCF-7 cells

The existence of GPR40 in the human breast cancer cell line MCF-7 is well known and its induction positively regulates cell growth activities^12^^,^^13^. The three compounds promoted MCF-7 cell proliferation. To avoid interferences with activation of RAR and RXR by tazarotenic acid and bezafibrate (this latter indirectly since the inhibition involved its obligate RXR partner), the cell line was exposed to specific antagonists as pre-treatment for 120 min. The EC_50_ values have been calculated for each compound and reported in Table 1.

## 4. Conclusions

A virtual screening approach based on the pharmacophoric generation and molecular docking simulations was applied for the discovery of new GPR40 allosteric agonists with activity on insulin release. Three compounds have been prioritised by the pharmacophore model that was used as a three-dimensional query to filter the DrugBank database of known drugs. Next, interactions of the best-ranked compounds with the allosteric binding site of GPR40, which also accommodates the agonist TAK-875, were identified by docking calculations. Tazarotenic acid, bezafibrate, and efaproxiral were found to induce cell growth and increase insulin secretion mediated by GPR40 activation. Based on these results, the compounds could be considered as promising starting points for the identification of new allosteric agonists of GPR40 whose development will be of pivotal importance for the treatment of type 2 diabetes and related diseases.

## References

[1] Park K. A review of computational drug repurposing. Transl Clin Pharmacol 2019;27:59–63. 32055582 10.12793/tcp.2019.27.2.59 PMC6989243

[2] Baker NC, Ekins S, Williams AJ, Tropsha A. A bibliometric review of drug repurposing. Drug Discov Today 2018;23:661–72. 29330123 10.1016/j.drudis.2018.01.018 PMC5963941

[3] Kristinsson H, Smith DM, Bergsten P, Sargsyan E. FFAR1 is involved in both the acute and chronic effects of palmitate on insulin secretion. Endocrinology 2013;154:4078–88. 24035997 10.1210/en.2013-1352

[4] Carullo G, Perri M, Manetti F, et al. Quercetin-3-oleoyl derivatives as new GPR40 agonists: molecular docking studies and functional evaluation. Bioorg Med Chem Lett 2019;29:1761–4. 31104992 10.1016/j.bmcl.2019.05.018

[5] Cione E, Plastina P, Pingitore A, et al. Capsaicin analogues derived from n-3 polyunsaturated fatty acids (PUFAs) reduce inflammatory activity of macrophages and stimulate insulin secretion by β-cells in vitro. Nutrients 2019;11:915–29. 10.3390/nu11040915 PMC6520993 31022842

[6] Yamada H, Yoshida M, Ito K, et al. Potentiation of glucose-stimulated insulin secretion by the GPR40-PLC-TRPC pathway in pancreatic β-cells. Sci Rep 2016;6:25912–21. 27180622 10.1038/srep25912 PMC4867641

[7] Hamdouchi C, Kahl SD, Lewis AP, et al. The discovery, preclinical, and early clinical development of potent and selective GPR40 agonists for the treatment of type 2 diabetes mellitus (LY2881835, LY2922083, and LY2922470). J Med Chem 2016;59:10891–916. 27749056 10.1021/acs.jmedchem.6b00892

[8] Krug AW, Vaddady P, Railkar RA, et al. Leveraging a clinical phase Ib proof-of-concept study for the GPR40 agonist MK-8666 in patients with type 2 diabetes for model-informed phase II dose selection. Clin Transl Sci 2017;10:404–11. 28727908 10.1111/cts.12479 PMC5593169

[9] Abdel-Magid AF. GPR40 receptor agonists for the treatment of type 2 diabetes and related diseases. ACS Med Chem Lett 2018;9:870–1. 30258532 10.1021/acsmedchemlett.8b00343 PMC6142065

[10] Negoro N, Sasaki S, Mikami S, et al. Discovery of TAK-875: a potent, selective, and orally bioavailable GPR40 agonist. ACS Med Chem Lett 2010;1:290–4. 24900210 10.1021/ml1000855 PMC4007909

[11] Milligan G, Shimpukade B, Ulven T, Hudson BD. Complex pharmacology of free fatty acid receptors. Chem Rev 2017;117:67–110. 27299848 10.1021/acs.chemrev.6b00056

[12] Yonezawa T, Katoh K, Obara Y. Existence of GPR40 functioning in a human breast cancer cell line, MCF-7. Biochem Biophys Res Commun 2004;314:805–9. 14741707 10.1016/j.bbrc.2003.12.175

[13] Fukushima K, Takahashi K, Kusaka M, et al. Induction of GPR40 positively regulates cell motile and growth activities in breast cancer MCF-7 cells. J Recept Signal Transduct Res 2018;38:311–5. 30111226 10.1080/10799893.2018.1494742

[14] Ebisawa M, Ohta K, Kawachi E, et al. Novel retinoidal tropolone derivatives. Bioisosteric relationship of tropolone ring with benzoic acid moiety in retinoid structure. Chem Pharm Bull 2001;49:501–3. 10.1248/cpb.49.501 11310685

[15] Ebisawa M, Umemiya H, Ohta K, et al. Retinoid X receptor-antagonistic diazepinylbenzoic acids. Chem Pharm Bull 1999;47:1778–86. 10.1248/cpb.47.1778 10748721

[16] Takahashi B, Ohta K, Kawachi E, et al. Novel retinoid X receptor antagonists: specific inhibition of retinoid synergism in RXR-RAR heterodimer actions. J Med Chem 2002;45:3327–30. 12139443 10.1021/jm0255320

[17] Perri M, Caroleo MC, Liu N, et al. 9-cis Retinoic acid modulates myotrophin expression and its miR in physiological and pathophysiological cell models. Exp Cell Res 2017;354:25–30. 28300567 10.1016/j.yexcr.2017.03.022

[18] Kane MA, Folias AE, Pingitore A, et al. Identification of 9-cis-retinoic acid as a pancreas-specific autacoid that attenuates glucose-stimulated insulin secretion. Proc Natl Acad Sci USA 2010;107:21884–9. 21115832 10.1073/pnas.1008859107 PMC3003056

[19] Santoro M, Guido C, De Amicis F, et al. Bergapten induces metabolic reprogramming in breast cancer cells. Oncol Rep 2016;35:568–76. 26459431 10.3892/or.2015.4327

[20] Srivastava A, Yano J, Hirozane Y, et al. High-resolution structure of the human GPR40 receptor bound to allosteric agonist TAK-875. Nature 2014;513:124–7. 25043059 10.1038/nature13494

[21] Schrödinger, LLC, New York, NY, 2000. www.schrodinger.com

[22] Wishart DS, Feunang YD, Guo AC, et al. DrugBank 5.0: a major update to the DrugBank database for 2018. Nucleic Acids Res. 2018;46:D1074-82. 10.1093/nar/gkx1037 PMC5753335 29126136

[23] Badolato M, Carullo G, Perri M, et al. Quercetin/oleic acid-based G-protein-coupled receptor 40 ligands as new insulin secretion modulators. Future Med Chem 2017;9:1873–85. 29064290 10.4155/fmc-2017-0113

[24] Dixon SL, Smondyrev AM, Rao SN. PHASE: a novel approach to pharmacophore modeling and 3D database searching. Chem Biol Drug Des 2006;67:370–2. 16784462 10.1111/j.1747-0285.2006.00384.x

[25] Christiansen E, Urban C, Merten N, et al. Discovery of potent and selective agonists for the free fatty acid receptor 1 (FFA(1)/GPR40), a potential target for the treatment of type II diabetes. J Med Chem 2008;51:7061–4. 18947221 10.1021/jm8010178

[26] Li Z, Liu C, Yang J, et al. Design, synthesis and biological evaluation of novel FFA1/GPR40 agonists: new breakthrough in an old scaffold. Eur J Med Chem 2019;179:608–22. 31279294 10.1016/j.ejmech.2019.06.087

[27] Li Z, Ren Q, Wang X, et al. Discovery of HWL-088: a highly potent FFA1/GPR40 agonist bearing a phenoxyacetic acid scaffold. Bioorg Chem 2019;92:103209. 31487621 10.1016/j.bioorg.2019.103209

[28] Li Z, Wang X, Xu X, et al. Design, synthesis and biological activity of phenoxyacetic acid derivatives as novel free fatty acid receptor 1 agonists. Bioorg Med Chem 2015;23:7158–64. 26482570 10.1016/j.bmc.2015.10.011

[29] Li Z, Wang X, Xu X, et al. Design, synthesis and structure-activity relationship studies of novel phenoxyacetamide-based free fatty acid receptor 1 agonists for the treatment of type 2 diabetes. Bioorg Med Chem 2015;23:6666–72. 26420383 10.1016/j.bmc.2015.09.010

[30] Wang X, Zhao T, Yang B, et al. Synthesis and biological evaluation of phenoxyacetic acid derivatives as novel free fatty acid receptor 1 agonists. Bioorg Med Chem 2015;23:132–40. 25481394 10.1016/j.bmc.2014.11.016

[31] https://www.accessdata.fda.gov/drugsatfda_docs/label/2011/020600s008lbl.pdf [last accessed August 2020].

[32] Sidgiddi S, Allenby K, Okumu F, Gautam A. Bioavailability, pharmacokinetics, and transepidermal water loss of short contact tazarotene lotion 0.1% versus tazarotene (Tazorac®) cream 0.1%. J Clin Aesthet Dermatol 2019;12:16–24. PMC6777703 31641413

[33] https://www.drugbank.ca/drugs/DB08486 [last accessed August 2020].

[34] Suh J, Stea B, Tankel K, et al. In Results of the phase III ENRICH (RT-016) study of efaproxiral administered concurrent with whole brain radiotherapy (WBRT) in women with brain metastases from breast cancer. 50th Annual Meeting of ASTRO; 2008 September 21–25; Boston, MA.

[35] https://www.drugbank.ca/drugs/DB01393 [last accessed August 2020].

[36] https://clinicaltrials.gov/ct2/show/NCT04309773. [accessed August 2020].

[37] Ludvigsson JF, Bergquist A, Montgomery SM, Bahmanyar S. Risk of diabetes and cardiovascular disease in patients with primary sclerosing cholangitis. J Hepatol 2014;60:802–8. 24291242 10.1016/j.jhep.2013.11.017

